# Signaling Signatures and Functional Properties of Anti-Human CD28 Superagonistic Antibodies

**DOI:** 10.1371/journal.pone.0001708

**Published:** 2008-03-05

**Authors:** Zoe Waibler, Linda Y. Sender, Camilla Merten, Roland Hartig, Stefanie Kliche, Matthias Gunzer, Peter Reichardt, Ulrich Kalinke, Burkhart Schraven

**Affiliations:** 1 Paul-Ehrlich-Institut, Langen, Germany; 2 Institute of Molecular and Clinical Immunology, Otto-von-Guericke-University, Magdeburg, Germany; New York University School of Medicine, United States of America

## Abstract

Superagonistic CD28 antibodies (CD28SAs) activate T lymphocytes without concomitant perturbation of the TCR/CD3-complex. In rodents these reagents induce the preferential expansion of regulatory T cells and can be used for the treatment of autoimmune diseases. Unexpectedly, the humanized CD28 superagonist TGN1412 caused severe and life threatening adverse effects during a recently conducted phase I clinical trail. The underlying molecular mechanisms are as yet unclear. We show that TGN1412 as well as the commercially available CD28 superagonist ANC28.1 induce a delayed but extremely sustained calcium response in human naïve and memory CD4^+^ T cells but not in cynomolgus T lymphocytes. The sustained Ca^++^-signal was associated with the activation of multiple intracellular signaling pathways and together these events culminated in the rapid de novo synthesis of high amounts of pro-inflammatory cytokines, most notably IFN-γ and TNF-α. Importantly, sustained transmembranous calcium flux, activation of Src-kinases as well as activation of PI3K were found to be absolutely required for CD28SA-mediated production of IFN-γ and IL-2. Collectively, our data suggest a molecular basis for the severe side effects caused by TGN1412 and impinge upon the relevance of non-human primates as preclinical models for reagents that are supposed to modify the function of human T cells.

## Introduction

According to the currently accepted model of T cell activation, two signals are required to fully activate resting naïve T lymphocytes. The primary signal is provided by the clonotypic T cell receptor (TCR) after recognition of antigen/MHC-complexes on the surface of antigen presenting cells. However, this signal by itself is not capable of fully activating T lymphocytes but has to be complemented by secondary signals which emerge from stimulation of so called co-stimulatory molecules [1], [2]. In mouse and human T cells the dimeric transmembrane glycoprotein CD28 represents the most important co-stimulatory molecule. Under physiological conditions CD28-derived signals alone are not capable of inducing T cell activation, whereas simultaneous engagement of the TCR and CD28 (e.g. by its natural ligands CD80 and CD86 which are expressed on mature antigen presenting cells) leads to activation of resting T lymphocytes (reviewed in [3], [4]).

Monoclonal antibodies (mAbs) directed to the extracellular domain of CD28 have been widely used during the last two decades to analyze CD28-mediated signaling pathways and to assess how CD28 facilitates activation and differentiation of murine, rat, and human T lymphocytes. Most recently a particular group of CD28 mAbs has been identified which is capable of activating T cells without the need for additional engagement of the TCR/CD3-complex [5]–[7]. These antibodies have collectively been termed mitogenic CD28 antibodies or CD28 superagonists. While conventional CD28 mAbs bind CD28 close to the binding site of the natural CD28 ligands, CD80 and CD86, CD28 superagonists bind to a laterally exposed loop within the extracellular domain of CD28 [8]. The particular binding topology of superagonistic CD28 antibodies (CD28SAs) might be responsible for their mitogenic potential.

A number of detailed biochemical studies in rat and mice addressed the question how CD28SA-mediated signaling is organized on the molecular level [5], [6], [8]–[13]. The emerged data can be summarized as follows: (i) the signaling capacity of CD28SAs depends on the expression of a functional TCR/CD3/ζ-complex; (ii) CD28SA-stimulation does not lead to detectable phosphorylation/activation of the TCRζ chain or the proximal TCR-effector molecules ZAP70 and LAT, but still induces phosphorylation of the adapter protein SLP76 and the nucleotide exchange factor Vav (likely via the Tec-family protein tyrosine kinases Itk or Rlk); (iii) CD28SA-stimulation activates PLCγ1 (phospholipase Cγ1) and induces calcium flux, and (iv) CD28SA-stimulation activates PKC θ (protein kinase C θ) as well as the transcription factors NF-κB, NF-ATc1, and GATA-3.

Studies in rat and mice have also shown that CD28 superagonists preferentially induce the expansion of regulatory T cells and therefore suggested that these antibodies can be used for the treatment of autoimmune diseases such as experimental autoimmune encephalomyelitis [13]–[20]. Based on the promising data in rodents, it was hypothesized that CD28SAs might also be applicable for the treatment of human autoimmune disorders. However, when applied to healthy volunteers during a phase I clinical trial performed on March 13^th^, 2006 in London, UK, the humanized CD28 superagonist TGN1412 showed unexpected serious adverse events. These were associated with the induction of a cytokine storm, i.e. the release of high amounts of pro-inflammatory cytokines, most notably TNF-α and IFN-γ [21].

The molecular basis for the unexpected response upon treatment with the CD28 superagonist TGN1412 is as yet unclear. To shed light on this question, we here addressed membrane proximal signaling events in human T cells upon stimulation with two different CD28 superagonists. We show that, despite complete conservation of the CD28 extracellular and cytoplasmic domains, TGN1412 and a commercially available CD28SA, ANC28.1/5D10, induced a delayed but extremely sustained calcium response in human, but not in cynomolgus and rhesus monkey T cells. Biochemical analyses further revealed that both CD28SAs strongly activated a number of major T cell signaling pathways in human T cells. Together these signals culminate in the in vitro production of high amounts of IFN-γ, TNF-α, and other pro-inflammatory cytokines.

Our experiments contribute to the understanding of the particular pharmacologic properties of human CD28 superagonists and further suggest that currently available animal models do not necessarily correctly reproduce critical signaling mechanisms of human T cells.

## Results

### Mitogenicity of anti-human CD28 superagonist ANC28.1

**Figure 1:**
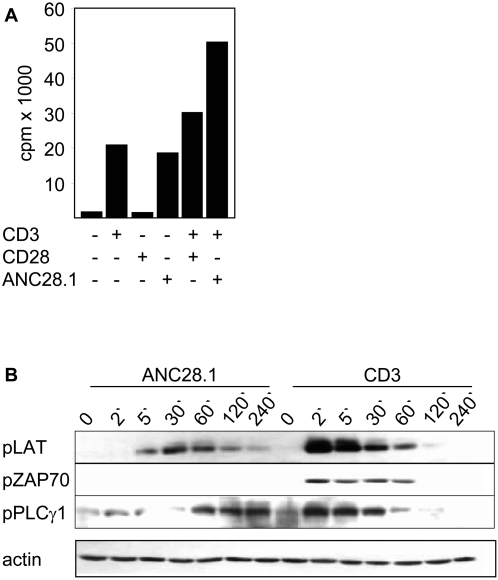
The CD28 superagonist ANC28.1 induces polyclonal T cell activation in vitro and leads to ZAP70 independent phosphorylation of LAT and PLCγ1. (A) 5×10^4^ freshly prepared human T cells per well were seeded in 96-well plates and treated with the indicated combinations of antibodies or were incubated in medium as a control. 72 hours after stimulation, cultures were pulsed with ^3^H-thymidine for 6 hours and harvested thereafter. Note that ANC28.1 only induces polyclonal T cell proliferation when applied in soluble form (in this case 10 µg/ml of soluble ANC28.1 were crosslinked in solution with 20 µg/ml of polyclonal goat-anti-mouse antiserum). All other stimulations (including stimulation of T cells with CD3ε mAb and ANC28.1; very right bar in Fig. 1A) were performed in wells that were pre-coated with polyclonal goat anti-mouse antiserum. Shown data are representative for at least 6 independently performed experiments. (B) 1×10^6^ freshly prepared human T cells/lane were stimulated in solution with 10 µg/ml ANC28.1 followed by crosslinking with 20 µg/ml polyclonal goat anti-mouse antiserum for the indicated periods of time. As a control, cells were activated with a 1∶50 v/v dilution of ascites fluid of CD3ε mAb MEM92 (IgM) or were left untreated. Cell lysates were processed for western blotting using the indicated phosphospecific antibodies (Y^319^ of ZAP70, Y^171^ of LAT, and Y^783^ of PLCγ1). Western blotting using an anti-actin antibody was performed as loading control.

By definition, CD28 superagonists induce polyclonal T cell activation in vitro without the need of concomitant stimulation of the TCR/CD3 complex [5], [7]. We assessed the mitogenicity of a commercially available CD28 superagonist (ANC28.1). In contrast to stimulation of human T cells with the conventional CD28 antibody 248.23.2 [22], ANC28.1 was able to induce T cell proliferation without additional stimulation via CD3 (Fig. 1A), thus fulfilling the criteria of a CD28 superagonist. Note that ANC28.1 still exerted costimulatory properties as it strongly augmented the proliferative response of T cells stimulated with CD3 antibody (Fig. 1A).

Previously, several groups have assessed the signaling events induced by anti-rat or anti-mouse CD28 superagonistic antibodies [5], [6], [8], [11]–[13]. In contrast, only few data are available regarding the signaling properties of anti-human CD28 superagonists. To explore the signaling properties of ANC28.1, phosphospecific western blots were performed (Fig. 1B). The transmembrane adapter protein LAT (Linker for Activation of T cells) represents a 35 kDa polypeptide that is essential for T cell activation [23], [24]. Upon TCR-mediated phosphorylation of four critical tyrosine residues, LAT assembles a multi-component signaling complex consisting of the cytosolic adapter proteins Gads (Grb2-related adaptor downstream of Shc) and SLP76 (SH2 domain containing leucocyte specific phosphoprotein of 76 kDa), the Tec-family protein tyrosine kinase Itk (Inducible T-cell kinase), and PLCγ1 (Phospholipase Cγ1; reviewed in [25]). This complex is responsible for the rise in intracellular calcium upon stimulation of the TCR. We first employed a phosphospecific antibody that detects one of the major tyrosine phosphorylation sites within LAT, Y^171^. As shown in Fig. 1B, upper panel, ANC28.1 induced a weak but clearly detectable phosphorylation of Y^171^. Similar data were obtained when the phosphorylation status of Y^136^, one of several additional phosphorylation sites of LAT, was assessed (data not shown). Hence, in contrast to rat T cells, anti-human CD28 superagonist ANC28.1 induced a weak but clearly detectable phosphorylation of LAT. Interestingly and in line with previous data obtained in human and rat T cells [8], [12], ANC28.1 stimulation did not result in a detectable phosphorylation (a sign for activation) of the protein tyrosine kinase ZAP70 which is believed to be primarily responsible for LAT phosphorylation upon TCR-stimulation (Fig. 1B, middle panel).

Upon engagement of the TCR/CD3-complex, phosphorylated LAT facilitates the activation of PLCγ1 which in turn generates the second messenger molecules IP3 (Inositol-tris-phosphate) and DAG (Diacyloglycerol; reviewed in [25]). To assess whether the moderate phosphorylation of LAT induced by ANC28.1 was sufficient to activate PLCγ1, we investigated the phosphorylation status of Y^783^ of PLCγ1 which correlates with its enzymatic activity [26]. The lower panel of Fig. 1B demonstrates that similar to rat T cells [11] ANC28.1 induced a weaker and delayed but clearly sustained activation of PLCγ1 when compared to stimulation of human T cells with CD3 mAb. Thus, ANC28.1 induced a phosphorylation pattern that was different from the one obtained when human T cells were activated via CD3.

### *Sustained calcium flux upon T cell stimulation with superagonistic* ANC28.1

**Figure 2 pone-0001708-g002:**
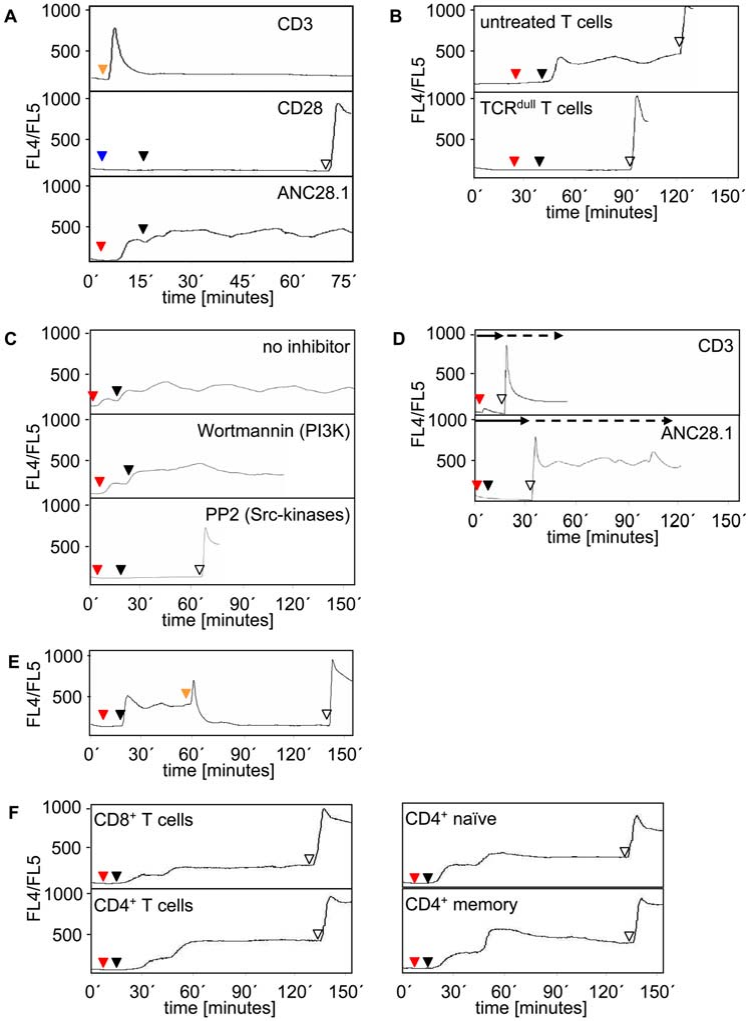
Induction of sustained calcium flux by human CD28 superagonistic antibody ANC28.1. (A) 1×10^6^ freshly prepared Indo-1 loaded human T cells were stimulated with either a 1∶50 v/v dilution of ascites fluid of CD3 mAb MEM92 (orange triangle), 10 µg/ml conventional CD28 mAb (CD28.2; blue triangle) followed by crosslinking with 20 µg/ml polyclonal goat anti-mouse antiserum (black triangle) or 10 µg/ml ANC28.1 (red triangle) followed by crosslinking with 20 µg/ml polyclonal goat anti-mouse antiserum (black triangle). Induction of the Ca^++^-response was monitored for approximately 75 minutes. Note that using another conventional anti-CD28 mAb (248.23.2; with or without crosslinking) no Ca^++^-flux was induced as well (data not shown). (B) Untreated (untreated T cells) or 2AD2A2 pre-treated (TCR^dull^ T cells) human T cells were stimulated with ANC28.1 (red triangles) and subsequently crosslinked (black triangles) as described in (A) and induction of the Ca^++^-response was monitored for the indicated periods of time. To confirm proper loading of the cells with Indo-1, the Ca^++^-ionophore Ionomycin (10 µg/ml) was added at the end of the experiment (unfilled triangle). (C) Cells were treated with ANC28.1 (red triangles) followed by crosslinking (black triangles) as described in (A) in the absence (no inhibitor) or presence of the PI3-kinase inhibitor Wortmannin (0.2 µM) or the Src-kinase inhibitor PP2 (10 µM). (D) CD3 (orange triangle) or ANC28.1 (red triangle) stimulation plus crosslinking (black triangle) was performed as described in (A) in culture medium that was supplemented with 1 mM EGTA to chelate extracellular Ca^++^ ions (solid arrow). At the indicated time points CaCl_2_ was added at a final concentration of 2 mM (dashed arrow). (E) Cells were treated with ANC28.1 as described in Fig. 1A. Additionally, at about 1 h of Ca-flux, a 1∶50 v/v dilution of ascites fluid of CD3 mAb MEM92 (orange triangle) was added. (F) CD8^+^, CD4^+^, CD4^+^/CD45RA^+^ naïve, and CD4^+^/CD45RO^+^ memory T lymphocytes were treated with ANC28.1 as described in (A) and induction of the Ca^++^-response was monitored for approximately 150 minutes.

The different activation kinetics of PLCγ1 produced by CD3 vs. CD28 superagonist ANC28.1 were translated into corresponding Ca^++^-responses (Fig. 2A). Thus, independently of the concentrations used the CD3ε mAbs MEM92 (Fig. 2A, upper panel and Fig. S1) and OKT3 (Fig. S1 and [27]) induced a rapid, strong, and transient rise in intracellular Ca^++^ whereas the CD28SA-mediated Ca^++^-signal started delayed and was of lower intensity (Fig. 2A, lower panel). Surprisingly, an extended analysis revealed that the CD28SA-mediated Ca^++^-signal had an extremely sustained kinetics (Fig. 2A–2F) that lasted for more than six hours without significant decrease (Fig. S2).

The flow cytometric data obtained with Indo-1 labeled T cells were confirmed by live time video microscopy of T cells loaded with the calcium sensitive dye Fura2 (Movie S1 and Movie S2). Note that stimulation of the same cells with two conventional CD28 mAbs (CD28.2, Fig. 2A, middle panel and 248.23.2, data not shown) did not induce any detectable Ca^++^-signal either with or without crosslinking.

Downmodulation of the TCR by treatment of T cells with the CD3ε mAb 2AD2A2 [28] (Fig. 2B, TCR^dull^ T cells) or pre-treatment of T cells with the Src-kinase inhibitor PP2 (Fig. 2C, lower panel) completely abrogated the ANC28.1-mediated calcium response, whereas pharmacologic inhibition of PI3K by Wortmannin had almost no effect (Fig. 2C, middle panel). Hence, similar to the situation in rat T cells [11], [12] expression of a functional TCR and the activation of Src-kinases are required for ANC28.1-mediated calcium flux, whereas activation of PI3K appears to be dispensable. Further stimulation experiments in the presence of the calcium-chelator EGTA revealed that the ANC28.1-mediated Ca^++^-signal required the presence of extracellular calcium ions (Fig. 2D).

Importantly, after inducing the expected peak (compare Fig. 2A, upper panel), addition of a conventional CD3 mAb to CD28SA-stimulated T cells abrogated the sustained Ca^++^-flux (Fig. 2E). This might suggest that CD3 stimulation activates a negative feedback loop that is otherwise not activated upon CD28SA-stimulation.

We next assessed which T cell population preferentially responded to CD28SA stimulation. As expected from published work [29], [30] ANC28.1 induced a stronger Ca^++^-signal in CD4^+^ T cells compared to CD8^+^ T cells (Fig. 2F, left panels) whereas almost no differences were observed between CD4^+^/CD45RA^+^ naïve vs. CD4^+^/CD45RO^+^ memory T lymphocytes (Fig. 2F, right panels).

### Induction of T cell proliferation and sustained calcium flux by CD28 superagonist TGN1412

**Figure 3 pone-0001708-g003:**
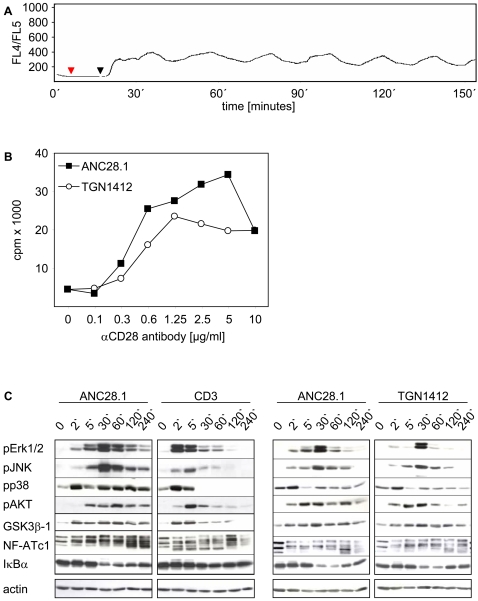
Sustained calcium flux, T cell proliferation, and T cell signaling upon TGN1412 treatment. (A) Freshly isolated human T cells were treated as described in Fig. 2 using the superagonistic CD28 specific mAb TGN1412 (10 µg/ml; red triangle). For crosslinking, 20 µg/ml of monoclonal mouse anti-human IgG_4_ was used (black triangle) and induction of the Ca^++^-response was monitored. (B) 5×10^4^ freshly prepared human T-cells/96-well were stimulated with the indicated concentrations of ANC28.1 or TGN1412 or were left untreated as a control. For crosslinking of ANC28.1, a polyclonal goat anti-mouse antiserum was used and crosslinking of TGN1412 was performed as described in (A). Concentrations for crosslinking were the following: 0.1–1.25 µg/ml primary Ab+2.5 µg/ml crosslinker; 2.5 µg/ml primary Ab+5 µg/ml crosslinker; 5 µg/ml primary Ab+10 µg/ml crosslinker; 10 µg/ml primary Ab+20 µg/ml crosslinker. 72 hours after stimulation, cultures were pulsed with ^3^H-thymidine for 6 hours, then harvested and proliferation analyzed by counts per minute [cpm]. (C) 1×10^6^ freshly prepared human T cells/lane were left untreated or were stimulated with 10 µg/ml ANC28.1 (first and third panel) followed by crosslinking with 20 µg/ml polyclonal goat anti-mouse antiserum or with 10 µg/ml TGN1412 (right panel) followed by crosslinking as described in (A) for the indicated periods of time. As a control, cells were activated with a 1∶50 dilution of ascites fluid of CD3 mAb MEM92 (second panel). Postnuclear lysates were processed for western blotting using the indicated phosphospecific antibodies. Western blotting using an anti-actin antibody was performed as loading control.

To exclude the possibility that the induction of a sustained calcium signal was a particular property of ANC28.1, we repeated the experiment shown in Fig. 2A using the superagonistic CD28-specific antibody TGN1412. Fig. 3A depicts that TGN1412 generated a similarly shaped calcium signature as antibody ANC28.1 although it induced a calcium response only upon crosslinking. In addition, the magnitude of the calcium response elicited by TGN1412 was generally lower than that of ANC28.1. The weaker signaling properties of TGN1412 compared to the ANC28.1 were also reflected by a generally lower mitogenic potential of TGN1412 (Fig. 3B).

### Intracellular signaling events induced by ANC28.1 and TGN1412

Given the sustained calcium response induced by ANC28.1 and TGN1412 we next investigated the consequences of CD28SA stimulation on downstream signaling events using reporter antibodies that monitor the activity of key signaling molecules involved in T cell activation. The first and third panels of Fig. 3C demonstrate that mAb ANC28.1 induced a delayed, strong, and sustained activation of all signaling events we investigated (Erk1/2, JNK, p38, AKT, GSK3β-1, NF-ATc1, and IκBα). Of note, ANC28.1 induced an even stronger activation of JNK, p38, AKT, and IκBα compared to CD3 mAb (second panel), whereas activation of Erk1/2 and dephosphorylation of the transcription factor NF-ATc1 were less pronounced. Similar results as for ANC28.1 were obtained when cells were stimulated with TGN1412 (Fig. 3C, right panel). However, in line with the lower magnitude of Ca^++^-signaling and the weaker induction of a proliferative response, the phosphorylation signals induced by TGN1412 were generally less pronounced than those induced by ANC28.1. In multiple experiments, both ANC28.1 and TGN1412 induced a sharp initial rise of p38 phosphorylation which transiently dropped after 5 minutes of stimulation. The reason for this particular phosphorylation signature is yet unknown. Note that a conventional CD28 antibody only induced the known activation of the AKT and the JNK pathways (data not shown and [31]). In summary, both human CD28 superagonists not only produced a sustained induction of Ca^++^-flux but also an activation of multiple intracellular signaling pathways.

### In vitro cytokine production upon conventional vs. superagonistic human T cell stimulation

**Figure 4:**
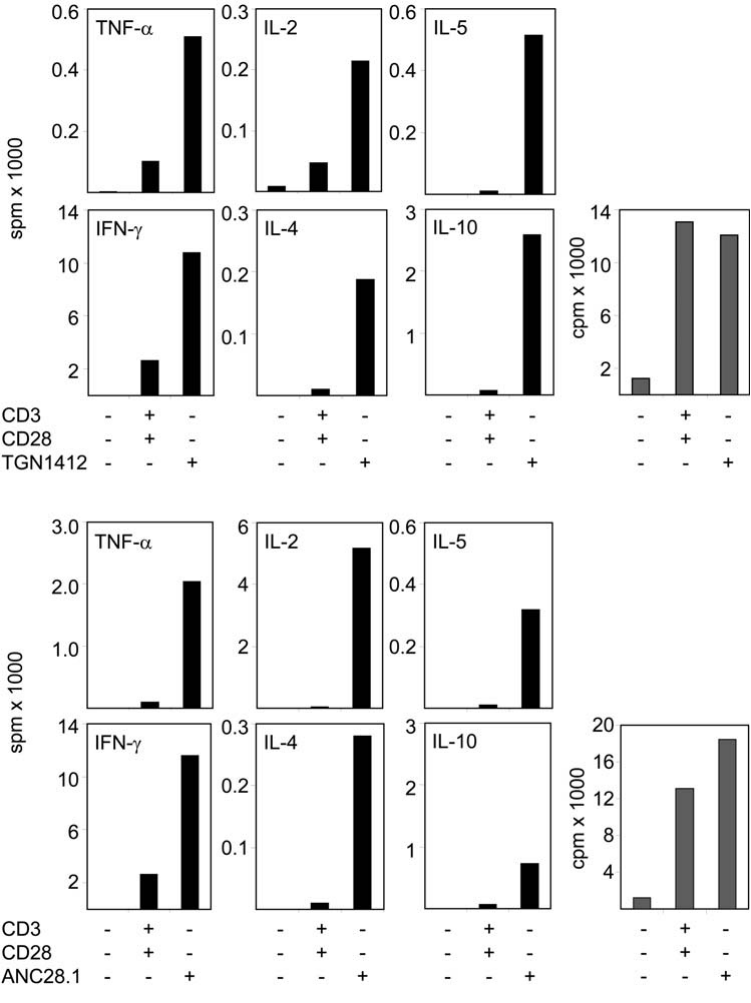
Cytokine production upon conventional and superagonistic CD28 stimulation of human T cells. 5×10^4^ freshly prepared human T-cells/96-well were stimulated with the indicated combination of antibodies as described in Fig. 1A or were left untreated as controls. At 48 hours 50 µl culture supernatant was removed from each well. Supernatants obtained from 3 wells were pooled and cytokine content was determined using the Cytokine Bead Array from BD in signals per minute [spm] (black bars). Additionally, induction of T cell proliferation was monitored upon treatment with the indicated antibody combinations (gray bars). 72 hours after stimulation, cultures were pulsed with ^3^H-thymidine for 6 hours, then harvested and proliferation analyzed by counts per minute [cpm]. Data represent the mean of 5 individual experiments.

Within hours after administration of TGN1412 to six healthy individuals, clinical symptoms became apparent that were consistent with a massive cytokine storm mediated by the release of TH1- and TH2-specific cytokines [21]. In line with previous data obtained using TGN1412 [32] Fig. 4 shows that also in vitro human T cells respond upon ANC28.1- or TGN1412-mediated stimulation with a strong production of high amounts of the pro-inflammatory cytokines IFN-γ, TNF-α, IL-2, -4, -5, and anti-inflammatory IL-10. In all experiments we observed that at the chosen concentration, TGN1412 was a better inducer of IL-10 than ANC28.1. In contrast (and in agreement with the generally stronger signals elicited by ANC28.1), the amounts of TNF-α, IFN-γ, IL-2, and IL-4 were higher upon stimulation with ANC28.1. Nevertheless, at concentrations inducing similar levels of proliferation, both CD28 superagonists induced the synthesis of significantly higher levels of cytokines compared to conventional CD3/CD28 co-stimulation. Hence, the activation of multiple signaling pathways upon CD28SA-mediated T cell activation is translated into a corresponding in vitro cytokine response.

**Figure 5:**
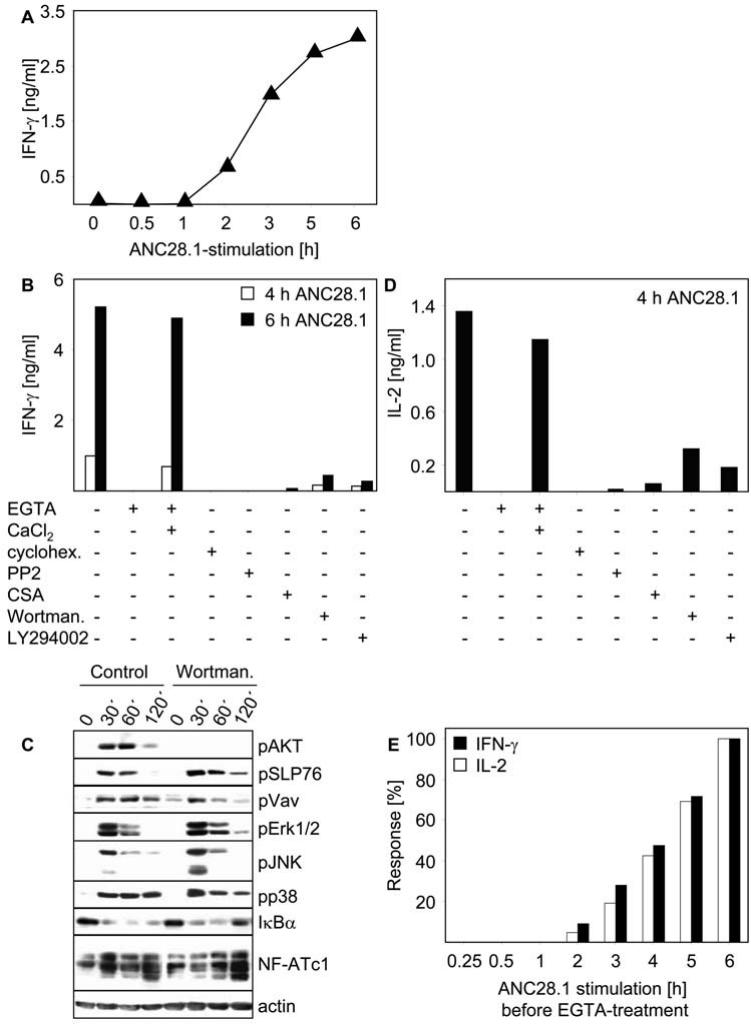
Analysis of the signaling pathways controlling CD28SA-mediated IFN-γ and IL-2 production. (A) Short term IFN-γ production upon CD28SA stimulation of T cells. 1×10^6^ T cells/200 µl were activated by CD28SA ANC28.1 as described for Figs. 1 [Fig pone-0001708-g002] [Fig pone-0001708-g003]–4. At the indicated time points 50 µl of the supernatants were removed, diluted 1∶3 v/v, and the concentration of secreted IFN-γ was determined using a commercially available ELISA. (B) Molecular requirements determining CD28SA-mediated IFN-γ production. T cells were pretreated for 30 minutes with the indicated substances prior to stimulation with ANC28.1. EGTA was used at 1 mM, CaCl_2_ at 2 mM, cycloheximide at 50 µg/ml, CSA at 150 ng/ml, PP2 at 10 µM, Wortmannin at 0.1 µM, and Ly294002 at 20 µM. At the indicated time points cytokine production was determined as described in (A). (C) 1×10^6^ freshly prepared human T cells/lane were stimulated with 10 µg/ml ANC28.1 followed by crosslinking with 20 µg/ml polyclonal goat anti-mouse antiserum with or without Wortmannin treatment. Western blotting was conducted as described in Fig. 3. Note that identical results were obtained when T cells were pretreated with LY294002 (data not shown). (D) Molecular requirements determining CD28SA-mediated IL-2 production. T cells were pretreated for 30 minutes with the indicated substances prior to stimulation with ANC28.1 as described in (B). 4 h after stimulation 50 µl of the supernatants were removed, diluted 1∶3 v/v, and the concentration of IL-2 was analyzed in supernatants by an ELISA. (E) CD28SA-mediated IFN-γ and IL-2 production requires sustained Ca^++^-flux. T cells were stimulated with ANC28.1 as described above. At 15′, 30′, 1 h, 2 h, 3 h, 4 h, and 5 h Ca^++^-flux was interrupted by addition of EGTA (1 mM) and the concentration of the cytokines in the supernatant was determined as described in (B) and (D). The concentrations of IL-2 and IFN-γ that were produced by ANC28.1-stimulated T cells following 6 hours of stimulation in the absence of EGTA were set to 100%. All data shown in Fig. 5 are representative for three independently performed experiments.

Fig. 5A depicts that similar to the situation in vivo, IFN-γ secretion occurred very rapidly after CD28SA stimulation. This allowed us to use pharmacological inhibitors to further dissect the molecular requirements leading to CD28SA-mediated IFN-γ production. Fig. 5B shows that incubation of T cells with cycloheximide, PP2, and Cyclosporin A (CSA) completely abrogated ANC28.1-mediated IFN-γ production. Hence, IFN-γ secretion upon CD28SA stimulation is due to de novo protein synthesis. Further, it requires activity of Src-kinases and involves the Calcineurin/NF-AT-mediated signaling pathway.

Similar to cycloheximide treatment, depletion of extracellular calcium ions by EGTA completely abrogated IFN-γ production after ANC28.1 stimulation. This finding shows that transmembranous calcium flux is mandatory for CD28SA-mediated induction of cytokine synthesis. Note that addition of extracellular Ca^++^ ions to EGTA-treated cells completely restored the CD28SA-induced IFN-γ response (Fig. 5B), excluding toxic EGTA effects.

Above we showed that the PI3K inhibitor Wortmannin did not influence CD28SA-mediated calcium flux (Fig. 2C). Similarly, neither Wortmannin nor a second PI3K inhibitor, LY294002, influenced CD28SA-mediated phosphorylation of SLP76, Vav, Erk, JNK, p38, the degradation of IKK or the dephosphorylation of NF-ATc1 (Fig. 5C), whereas both inhibitors completely abrogated CD28SA-mediated phosphorylation of AKT (upper panel in Fig. 5C). These data corroborate a recent report showing that CD28SAs still induce upregulation of CD69 in human T cells pretreated with Wortmannin or Ly294002 [33]. Surprisingly, both Wortmannin and Ly294002 induced an almost complete block of IFN-γ production after ANC28.1 stimulation (Fig. 5B). This suggests that PI3K or its downstream effector AKT [34], [35] is mandatory (although not sufficient) for CD28SA-mediated IFN-γ production.

We next assessed the mechanisms underlying CD28SA-mediated IL-2 induction. This was of particular interest because a recent report suggested that in rat T cells CD28SA induced IL-2 production might not require PI3K activity [12]. Fig. 5D shows that, similar to IFN-γ production, CD28SA-mediated IL-2 synthesis by human T cells was sensitive to treatment with EGTA, cycloheximide, CSA, and PP2. Furthermore, Wortmannin and LY294002 abrogated CD28SA-mediated IL-2 synthesis. Hence, in contrast to rat T cells, activation of PI3K was also mandatory for CD28SA induced IL-2 production by human T lymphocytes.

We finally assessed whether the sustained Ca^++^ signaling that is induced by ANC28.1 and TGN1412 (see Figs. 2 and 3) is required for cytokine production upon CD28SA-stimulation. To achieve this we inhibited transmembranous Ca^++^-flux at different time points after stimulation with ANC28.1 by the addition of EGTA and subsequently determined the concentration of IFN-γ and IL-2 in the culture supernatants. Fig. 5E depicts that the amounts of IFN-γ and IL-2 secreted by CD28SA-stimulated T cells directly correlated with the duration of CD28-mediated transmembranous Ca^++^-flux. Thus, sustained calcium flux is important for the strong production of IFN-γ and IL-2 upon CD28SA stimulation.

### Lack of a TGN1412-mediated calcium response in monkey T cells

Before being used in the London phase I clinical trial, TGN1412 had been applied to rhesus and cynomolgus monkeys without showing obvious severe adverse effects [36]. Furthermore, it was debated whether the extracellular domains of *Macaca fascicularis* (cynomolgus), *Macaca mulatta* (rhesus), and *Homo sapiens* CD28 show subtle differences on the protein level [37] [38]. To clarify the latter question we sequenced CD28 cDNAs obtained from 14 individual rhesus and 11 cynomolgus monkeys and compared the deduced amino acid sequences with the protein sequence of human CD28. Notably, this approach corroborated that the CD28 extracellular domains (as well as the cytoplasmic tails) are completely conserved between the three species (Fig. S3). Interestingly, in all 25 monkeys investigated we observed three non-conservative amino acid exchanges in the transmembrane region (Fig. S3). Furthermore, one non-conservative amino acid exchange was found within the leader sequence of the non-human primates.

**Figure 6:**
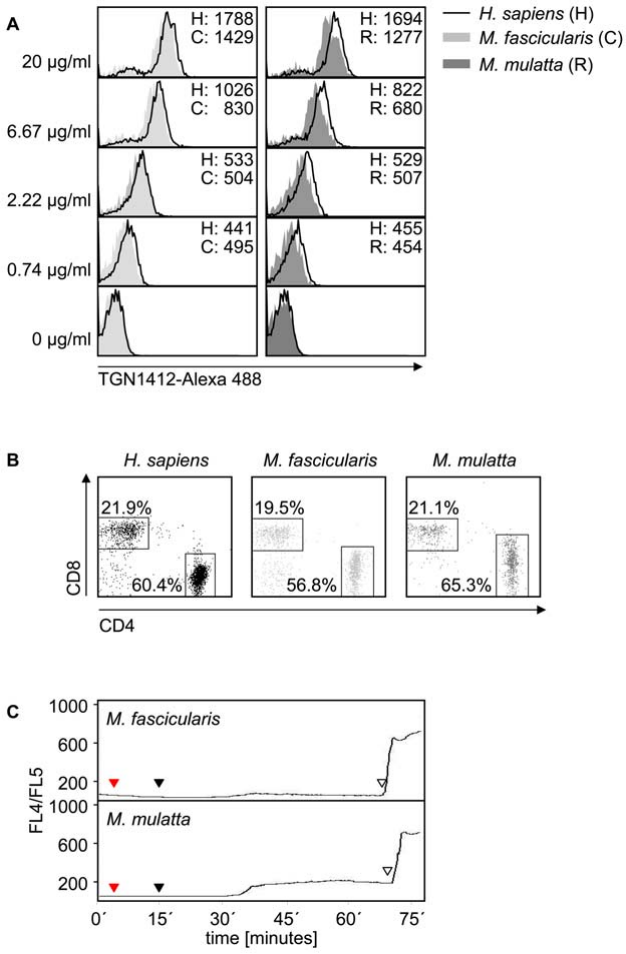
Despite similar TGN1412 binding, Macaca derived T cells show reduced calcium flux upon TGN1412 stimulation when compared to human T cells. (A) MACS purified CD3^+^ human T cells (stained with an anti-CD3-APC antibody) and CD3^+^ monkey T cells (stained with an anti-CD3-PE antibody) were mixed 1∶1 and then incubated with graded concentrations of Alexa 488 labeled TGN1412. Binding of TGN1412-Alexa 488 was monitored by FACS analysis. Numbers given indicate the mean fluorescence intensity of samples. (B) Ratio of CD4^+^ vs. CD8^+^ T cells (gated on CD3^+^ cells) of human and monkey blood samples was analyzed by FACS analyses. (C) 1×10^6^ freshly isolated Indo-1 loaded *M. fascicularis* and *M. mulatta* T cells were treated as described in Fig. 2 using the superagonistic CD28 specific mAb TGN1412 (10 µg/ml; red triangle). For crosslinking, 20 µg/ml monoclonal mouse anti-human IgG_4_ was used (black triangle) and induction of the Ca^++^-response was monitored. To confirm viability of cells and proper loading with Indo-1, Ionomycin (10 µg/ml) was added at the end of the experiment (unfilled triangle).

The complete conservation of the extracellular domains of CD28 across the investigated species was further reflected by similar TGN1412 binding of human CD3^+^ and non-human primate CD3^+^ T cells. A 1∶1 mixture of CD3^+^ human T cells (stained with an anti-CD3-APC antibody) and CD3^+^ non-human primate T cells (stained with an anti-CD3-PE antibody) derived from cynomolgus (Fig. 6A, left panel) or rhesus (Fig. 6A, right panel) monkeys, respectively, was incubated with Alexa 488 labeled TGN1412 at graded concentrations. Similar decoration of T cells from both species with TGN1412 indicated similar binding capacities of human and non-human primate CD28 to the antibody. Moreover, FACS analyses revealed a similar ratio of CD4^+^ vs. CD8^+^ T cells in blood samples of human and non-human origin (Fig. 6B).

Interestingly, despite similar TGN1412 binding, identical extracellular CD28 domains of all three species, and a similar ratio of CD4^+^ vs. CD8^+^ T cells in blood samples of human and non-human origin (Fig. 6 and Fig. S3), TGN1412 only induced a very low calcium signal in rhesus or cynomolgus T cells (Fig. 6C). Similar results were obtained with ANC28.1 (data not shown). Note that the profound differences in the Ca^++^-response between monkey and human T cells were also observed at higher concentrations of both antibodies (up to 20 µg/ml; not shown). Thus, despite comparable levels of CD28 expression and similar reactivity with TGN1412 only human T cells are capable of inducing a significant calcium response upon stimulation with CD28 superagonists.

## Discussion

The data shown in this report clearly demonstrate that two superagonistic CD28 mAbs, TGN1412 and ANC28.1, generate a remarkably sustained Ca^++^-response when applied to human T cells in vitro (Fig. 2 and 3). The prolonged Ca^++^-signal is preceded by a sustained activation of PLCγ1 (Fig. 1) and is downstream correlated with an activation of a number of different signaling pathways (Fig. 3) that are involved in the activation and differentiation of T lymphocytes. Together, these signals culminate in the production of a variety of pro-inflammatory cytokines, most notably IFN-γ and TNF-α (Fig. 4).

The strong and sustained Ca^++^-response elicited by the CD28SAs seems to be a particular property of the human system and it appears as if human T cells react more sensitive to CD28SA stimulation compared to rat and monkey T cells ([11] and Fig. 6). A sustained Ca^++^-response following CD28SA-stimulation has previously been described for rat T cells [11]. However, due to the low on-rate or the weak accessibility of the CD28 epitope detected by CD28SAs, the induction of Ca^++^-flux in rat T cells required antibody pre-incubation for several hours [11]. Similarly, the induction of proliferation by mitogenic rat CD28SA antibodies needs extensive crosslinking and we also observed that the binding of an anti-mouse CD28SA (D665) to the surface of murine T cells occurred with an extremely slow kinetics and was strongest upon overnight incubation on ice (data not shown).

In contrast, the binding of both TGN1412 and ANC28.1 to human T cells occurs rapidly and is almost indistinguishable from the binding of monoclonal antibodies directed to other cell surface receptors (data not shown). Hence, the binding of CD28SAs to human T cells occurs with much faster kinetics compared to mouse and rat CD28SAs. A faster on-rate (presumably combined with a slow off-rate) could explain why CD28SAs are capable of inducing sustained signaling in human T lymphocytes.

A number of previous studies had assessed the capability of conventional CD28 antibodies to induce Ca^++^-flux in Jurkat T cells and primary human T cells. These studies suggested that CD28-mediated Ca^++^-flux requires extracellular calcium ions and hence, is EGTA-sensitive [30]. However, they also suggested that CD28-mediated signals do not activate the PTK pathway [30], occur in the absence of TCR signals [30], do not lead to production of DAG [39], and also do not activate PKC. This appears not to be the case for the anti-human CD28SAs for the following reasons: (i) we show that (similar to rat T cells [12]) the Ca^++^-signal produced by ANC28.1 required expression of a functional TCR (Fig. 2B); (ii) anti-human CD28SA signaling was Src protein tyrosine kinase dependent as it could be blocked by PP2 (Fig. 2C); (iii) anti-human CD28SA-mediated signaling induced the induction of the DAG-dependent Ras/Raf/Erk pathway (Fig. 3C), and (iv) it led to activation of the IKK/NF-κB pathway (Fig. 3C) which is dependent on activation of PKC. Hence, despite some similarities, the signaling properties of conventional anti-human CD28 antibodies fundamentally differ from those of CD28 superagonists.

In agreement with data obtained in rat T cells [12], ANC28.1 stimulation did not result in a detectable tyrosine phosphorylation of the protein tyrosine kinase ZAP70 (Fig. 1B, middle panel) although ZAP70 is believed to be primarily responsible for LAT phosphorylation upon TCR-stimulation. At present it is unclear which protein kinase is responsible for LAT phosphorylation upon CD28 superagonistic stimulation of human T cells. Attractive candidates include the two Tec-family protein tyrosine kinases Itk and Rlk [12].

An important question that needs to be addressed in the future is why the calcium-signal induced by human superagonistic CD28 mAbs has such a dramatically prolonged kinetics. It is obvious from our study that the potency of CD28SAs to activate PLCγ1 and to induce Ca^++^-flux is lower than the potency of CD3 mAb. Thus, it is possible that the amount of IP3 generated upon triggering by CD28SAs suffices to activate membrane associated Ca^++^-channels, but that the overall Ca^++^ influx is below the threshold that is needed to close the channels. In line with this idea is our observation that the sustained calcium-response elicited by the CD28SAs can be stopped by administration of a CD3 mAb (Fig. 2E). However, further studies are required to elucidate the question how transmembrane flux is induced and maintained upon CD28SA-stimulation. Moreover, it will be important to determine the functional roles of the recently identified ORAI1 and STIM proteins in CD28SA-induced transmembrane Ca^++^-flux [40].

Our data show that CD28SA-stimulation leads to a strong and very rapid production of cytokines in vitro (Figs. 4 and 5). Indeed, IFN-γ was detectable in the culture supernatant already 90–120 minutes after CD28SA stimulation (Fig. 5A). Inhibition of protein synthesis by cycloheximide completely abrogated IFN-γ secretion (Fig. 5B) which strongly suggests that this event is not due to a release of the cytokine from intracellular stores but rather due to de novo IFN-γ synthesis. Furthermore, the production of IFN-γ was dependent on CD28SA-mediated Ca^++^-flux as pretreatment of the cells with EGTA completely blocked the response. Perhaps more importantly, our data show that the amounts of IFN-γ and IL-2 produced by CD28SA-treated cells directly depend on the duration of transmembranous Ca^++^-flux (Fig. 5E). Thus, it appears as if the large amounts of cytokines produced upon anti-human CD28SA-stimulation in vitro (and presumably also in vivo) are due to the sustained Ca^++^-signal generated by the CD28 superagonistic antibodies.

With regard to the induction of IL-2 synthesis the signaling properties of CD28 superagonists again appear to differ from those of conventional CD28 mAbs. Based on studies using cholera-toxin, it was suggested that conventional CD28 antibodies activate two functionally unrelated signaling pathways [22]. One of these pathways was found to be cholera-toxin sensitive, initiated transmembranous Ca^++^-flux, but did not influence IL-2 secretion whereas the other one was cholera-toxin insensitive and responsible for IL-2 production [22]. Both with regard to production of IL-2 and IFN-γ this mechanism does not hold true for signaling induced by CD28SAs. Indeed, we show that both the PTK/PLCγ/Ca^++^-pathway and the PI3-kinase pathway must operate simultaneously in order to allow IL-2 and IFN-γ synthesis. Whether this also applies for the production of other cytokines upon CD28SA stimulation requires further analysis. Moreover, despite costimulatory capacity, the same antibody that was used by Nunes et al. to dissect signaling events upon CD28-stimulation in Jurkat T cells (mAb 248.23.2 [22]) did not generate a detectable Ca^++^ signal in primary human T cells (data not shown). These differences might be due to the different cells that were investigated or to different CD28 antibody preparations (ascites fluid in [22] vs. cell culture supernatant in our study).

It is important to note that the signaling pathways induced by CD28SAs also seem to differ between rat and human T cells. A recent report suggested that IL-2 production upon CD28SA-stimulation of rat T cells is primarily mediated via the SLP76/Vav-module and does not require activation of PI3K [12]. The latter assumption was based on the observation that anti-rat CD28 superagonists do not induce phosphorylation of AKT and that mutation of the PI3K binding site within the cytoplasmic domain of CD28 did not ablate CD28SA-mediated IL-2 production [12]. However, our data show that CD28-mediated IFN-γ and IL-2 production required both transmembranous Ca^++^-flux and activation of PI3K (Fig. 5). Indeed, inhibition of PI3K blocked CD28SA-mediated IL-2 production in human T cells without influencing transmembranous Ca^++^-flux, phosphorylation of SLP76, Vav, Erk, JNK, p38 as well as the activation of the transcription factors NF-AT and NF-κB. Together these data suggest that anti-human CD28SA signaling involves at least two major signaling modules, one that depends on activity of PI3K and a second one that requires transmembranous Ca^++^-flux (note that treatment of T cells with EGTA did not substantially alter CD28SA-mediated activation of AKT (data not shown)). However, we certainly cannot exclude the possibility that CD28SA-stimulation activates a pool of PI3K that does not associate with CD28 upon CD28SA stimulation. Further, it will be important to investigate whether PI3K regulates the production of IL-2 and IFN-γ upon CD28SA stimulation in human T lymphocytes via its proposed downstream effector AKT [34], [35].

Differences between human and rodent T cell activation are also underlined by experiments analyzing Ca^++^-flux in CD4^+^ and CD8^+^ T cell subpopulations. It was shown previously that in mouse splenic T cells only CD4^+^ but not CD8^+^ T cells showed Ca^++^-flux upon CD28 triggering [29] whereas within human PBMCs also CD8^+^ cells were capable of Ca^++^-release upon CD28 stimulation [30]. In agreement with data shown in this study (Fig. 2F) responses by CD8^+^ T cells were less pronounced than those by CD4^+^ T cells [30].

It is still unclear how CD28SAs precisely activate the multiple intracellular signaling pathways at the level of the plasma membrane. Initially we thought that this might be due to formation of large CD28 clusters on the T cell surface. However, in line with a recent report [33] we did not see obvious differences in CD28 cluster formation upon CD28SA vs. CD28 stimulation by confocal laserscanning microscopy (data not shown). However, we cannot exclude the possibility that the assembly or the dynamics of the recently identified microclusters [41] differs between CD28 vs. CD28SA stimulation. It will be important to assess this possibility in the future.

Although we observed that CD28SA stimulation led to the activation of many intracellular signaling pathways it is important to note that CD28SAs do not fully activate human T cells. For example, we did not observe an activation of β1- or β2-integrins upon CD28SA stimulation and we also did not see formation of F-actin or induction of cell migration by CD28SAs (data not shown). Why these cellular events are not initiated upon CD28SA stimulation is unclear at present. One reason might be that the thresholds that are required to activate these pathways are higher than those regulating cytokine production.

The amino acid sequence of the extracellular domains of rhesus and cynomolgus CD28 are identical with the one of human CD28 (Fig. S3 and see genebank entries 111144662 and 112817616 for *Macaca mulatta* and 110611295, 111144664, and 110611297 for *Macaca fascicularis* as well as ref. [42]). This was also reflected by similar decoration of CD28 on T cells of human, *Macaca mulatta*, and *Macaca fascicularis* origin with Alexa 488 labeled TGN1412 (Fig. 6A). Intriguingly however, monkey T cells did not show Ca^++^-response upon CD28SA stimulation (Fig. 6C) as it was observed in human T cells. Unlike rodents, blood samples of non-human primates and humans show approximately 70% T cells in the lymphocyte gate and within the T cell population, non-human primates and humans show a similar ratio of CD4^+^ vs. CD8^+^ T cells (Fig. 6B and [43], [44]). It is also important to note that the non-human primates used in this study are not kept under specific pathogen free (SPF) conditions although they are regularly screened for infections with pathogens. Thus, the observation that TGN1412 stimulation induced Ca^++^-flux only in human but not in non-human primate T cells (Fig. 6C) can not be explained by significantly decreased numbers of CD4^+^ T cells in non-human primates or by a more naïve activation state of the T cells due to SPF housing conditions.

Importantly, a recent study showed that chimpanzee T cells are also less responsive towards in vitro stimulation than human T cells and it was suggested that this might be due to loss of Siglec expression on human T cells [45]. Siglecs are inhibitory receptors that are related to CD33 and it appears as if their expression is lost on T cells during evolution from monkey to human [45]. Hence, the lack of Siglec expression might explain why human T cells react more sensitive upon CD28SA stimulation compared to Macaca T cells.

Alternatively, the number/size and/or distribution of lipid rafts might differ between monkey and human T lymphocytes. Finally, three non-conservative amino acid exchanges are present within the transmembrane regions of Macaca monkey and human CD28 which might influence the lateral interactions between CD28 and other signaling molecules within the plasma membrane. Clearly, these points need to be investigated in the future.

Collectively, our functional and biochemical data provide a first explanation for the severe adverse effects induced upon TGN1412 administration to human beings. Further, they show that in spite of absence of toxicity signals in the Macaca model that is broadly considered as the golden standard for preclinical tests, a detailed in vitro analysis of human cells is still mandatory to reduce risks inherently related with first-in-man studies.

## Materials and Methods

### Animals

*Macaca mulatta* (rhesus monkey) and *Macaca fascicularis* (cynomolgus monkey) were kept under conventional conditions at the Central Animal Facility of the Paul-Ehrlich-Institut, Germany. The animals are regularly screened for infections according the recommendations of EUPRIM-Net including tuberculosis, alphaherpes virus, SIV, STLV, filovirus, and others. Experimental work was carried out in compliance with regulations of German animal welfare.

### T cell purification

Peripheral blood mononuclear cells (PBMC) were isolated by Ficoll gradient (Biochrom) centrifugation of heparinized blood collected from healthy volunteers. Human T cells were further purified by non-T cell depletion using the Pan T cell isolation kit II (Miltenyi Biotec). For monkey T cell enrichment, monkey blood was Ficoll purified using individually adjusted Ficoll dilutions (between 95 and 100%). Then T cells were MACS purified using the Pan T cell isolation kit for non-human primates (Miltenyi Biotec). Human CD8^+^, CD4^+^, CD4^+^/CD45RA^+^ naïve, and CD4^+^/CD45RO^+^ memory T lymphocytes were purified using the CD4^+^/CD8^+^ T cell isolation kit and the CD4^+^ naïve/memory T cell isolation kit (Miltenyi Biotec).

### Antibodies

For the different applications the following antibodies were used: for cytokine release assays and proliferation assays the CD3ε monoclonal antibody OKT-3 (purchased from ATCC) was used as hybridoma supernatant. Stimulations for western blotting experiments and calcium release assays were performed using the CD3ε monoclonal antibody MEM92, kindly provided by Dr. Vaclav Horejsi, Prague Academy of Sciences, Prague, Czech Republic. For conventional co-stimulation, hybridoma supernatant of the CD28 monoclonal antibody 248.23.2 [22], [46] or commercially available CD28.2 (Biosciences) was used. The superagonistic CD28 antibody ANC28.1/5D10 was obtained from Ancell (referred to as ANC28.1 throughout the text) and humanized superagonistic CD28 antibody TGN1412 was a gift from Thomas Hanke.

### In vitro proliferation assays

96-well round-bottomed tissue culture plates (Costar) were coated with a 1∶400 v/v dilution of polyclonal goat-anti-mouse antiserum (specific for IgG and IgM, 50 µl/well). Plates were then washed three times with phosphate buffered saline (Seromed) and either 100 µl of CD3ε mAb OKT-3 (1∶2 v/v dilution of hybridoma culture supernatant), 100 µl of conventional CD28 mAb 248.23.2 (1∶2 v/v dilution of hybridoma culture supernatant) or 100 µl of a 1∶1 mixture of OKT-3 and 248.23.2 culture supernatants were added. For CD3xANC28.1 induced proliferation 100 µl of a 1∶2 v/v dilution of hybridoma culture supernatant of CD3ε mAb OKT-3 were supplemented with 10 µg/ml of ANC28.1 were used. Alternatively, T cells were incubated with the indicated concentrations of ANC28.1 or TGN1412 and crosslinked with goat anti-mouse (IgG+IgM) polyclonal serum (Dianova) or purified mouse anti-human IgG_4_ monoclonal antibody (BD Pharmingen), respectively. For T cell proliferation experiments, 5×10^4^ T cells/well were added in a final volume of 200 µl. [3H]-thymidine (0.3 µCi/well; specific activity 50 Ci/mmol) was added for the last 8–10 hours of the three day incubation and cells were harvested using a PHD cell harvester. Thymidine incorporation was measured by liquid scintillation in counts per minute [cpm].

### In vitro cytokine production

Cells were stimulated in vitro in triplicates as described for T cell proliferation. Approximately 48 hours after onset of the stimulation 50 µl of culture supernatant was removed from each well. Triplicate supernatants were pooled and 50 µl of each pool was then used in the TH1/TH2/Inflammatory Cytometric Bead Arrays (CBA) from Becton Dickenson according to the manufacturer's recommendation. Cytokine expression is shown in signals per minute [spm]. Data shown represent the mean of at least three individual experiments.

To determine the concentration of IFN-γ and IL-2 after short term stimulation of T cells, the cell suspension was adjusted to 1×10^5^ T cells/200 µl. Cells were stimulated as described above for the indicated periods of time. Subsequently, 50 µl culture supernatant was removed and diluted 1∶3 v/v in culture medium. The concentrations of IFN-γ and IL-2 were determined using the Quantikine ELISA system from R&D Systems according to the manufacturer's recommendation. To assess the molecular requirements for CD28SA-mediated IFN-γ and IL-2 production the following inhibitors were used: EGTA (1 mM), CaCl_2_ (2 mM), cycloheximide (50 µg/ml; Sigma), CSA (150 ng/ml; Calbiochem), PP2 (10 µM; Calbiochem), Wortmannin (0.1 µM; Calbiochem), and Ly294002 (20 µM; Calbiochem).

### Western blotting

T cells were lysed in buffer containing 1% lauryl maltoside (N-dodecyl β-maltoside), 1% NP-40, 1 mM Na_3_VO_4_, 1 mM PMSF, 10 mM NaF, 10 mM EDTA, 50 mM Tris pH 7.5, and 150 mM NaCl. Postnuclear lysates were separated by SDS-PAGE and transferred onto nitrocellulose membranes. Membranes were probed with the indicated primary antibodies and the appropriate HRP-conjugated secondary antibodies (Dianova) and developed using the ECL detection system (Amersham Pharmacia). The following antibodies were used for western blotting in this study: anti-pErk1/2 (Thr^202^/Tyr^234^), anti-pZAP70 (Y^319^), anti-pLAT (Y^171^), anti-pJNK (Thr^183/185^), anti-pp38 (Thr^180/182^), anti-IκBα, anti-AKT, and anti-GSK3β-1 (all from Cell signaling), anti-PLCγ1 (Y^783^, Santa Cruz), anti-NF-ATc1 (Alexis/Axxora), anti-β-actin (Sigma), anti-pVav (Y^174^ Santa Cruz), and anti-pSLP76 (BD Biosciences).

### Calcium measurements

Purified T cells (2×10^7^ cells/ml) in RPMI medium (phenol-red free; Invitrogen) containing 10% FCS were loaded with 5 µg/ml Indo-1-AM (Molecular Probes) at 37°C for 45 min. After washing, cells were incubated in RPMI medium supplemented with 10% FCS (phenol-red free) at 37°C for an additional 45 min. The measurement was performed on a FACSort™ flow cytometer (Becton Dickinson). The kinetics of the data was analyzed with FlowJo software (TreeStar).

### Sequencing

Blood was taken from 14 individuals *Macaca mulatta* (rhesus monkey) and 11 individuals *Macaca fascicularis* (cynomolgus monkey). The animals analyzed belong to different families and were from different origins to avoid inbreeding effects. RNA from blood cells was isolated using Paxgene RNA Blood Kit (Qiagen) in combination with Paxgene Blood RNA tubes (Preanalytix). RNA was incubated with DNase I (Roche) for 15 min at 37°C and cDNA was prepared by using SuperScript II (Invitrogen) according to manufacturer's instructions. RT-PCR with RNA as template using GAPDH-specific primers confirmed the absence of genomic DNA within all samples and controls with no template confirmed specificity. Primers used for amplification were the following (sequences listed in 5′ to 3′ orientation): GAPDH: ACCACAGTCCATGCCATCAC and TCCACCACCCTGTTGCTGTA; CD28: CTCACACTTCGGGTTCCTCG and GGTCATTTCCTATCCAGAGC. RT-PCR fragments were sequenced both forward and reverse (MWG). Of note, there were individual differences in the nucleotide sequences not resulting in exchanged amino acids.

### FACS Analysis

For FACS analysis TGN1412 was labeled using the Alexa Flour®488 monoclonal labeling kit (Invitrogen/Molecular Probes) according to the manufacturer's instructions. Human and monkey blood was stained with TGN1412-Alexa Flour®488 at the indicated concentrations in a total volume of 50 µl. Human and monkey cells were stained using the following antibodies: anti-CD3-PE, -APC or -FITC, anti-CD4-PE, anti-CD8-FITC or -PerCP (all from BD). After washing with FACS-buffer, samples were incubated with blood lysing buffer (BD Pharm Lyse™) for 2–3 hours at 4°C and washed again. Cells were analyzed via Flow cytometry (LSR™ II; BD) and evaluated with DIVA® software. One representative experiment out of three comparable experiments is shown.

## Supporting Information

Figure S1 (1.76 MB TIF)

Figure S2 (3.22 MB TIF)

Figure S3 (0.51 MB TIF)

Movie S1 (4.45 MB MOV)

Movie S2 (2.67 MB MOV)
