# Supplementary figures and images for: Signaling Signatures and Functional Properties of Anti-Human CD28 Superagonistic Antibodies
Source: PLoS One. 2008 Mar 5;3(3):e1708. doi: 10.1371/journal.pone.0001708 (PMC2246163; doi:10.1371/journal.pone.0001708)

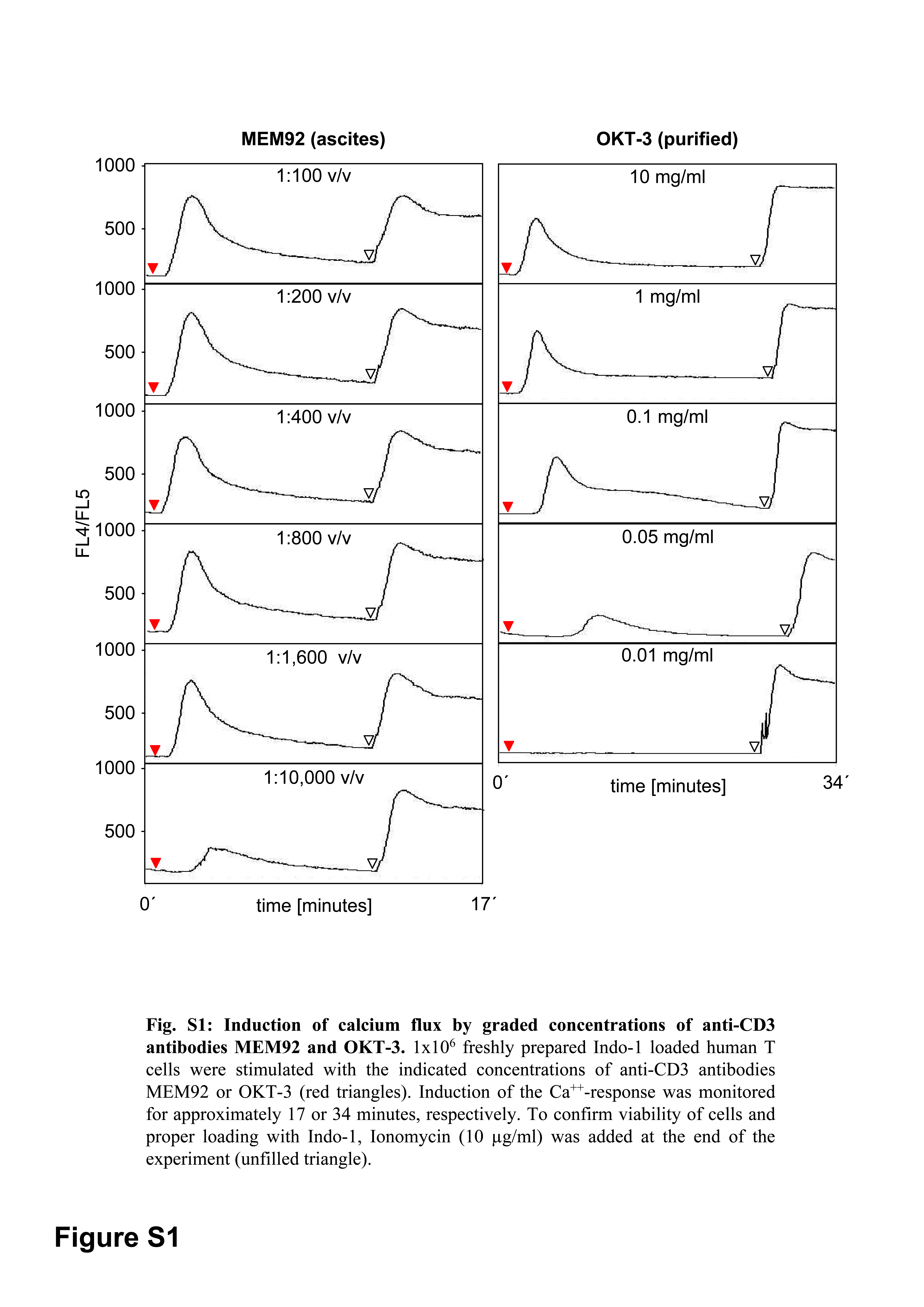

Supplement: Figure S1 — (1.76 MB TIF) [file pone.0001708.s001.tif]

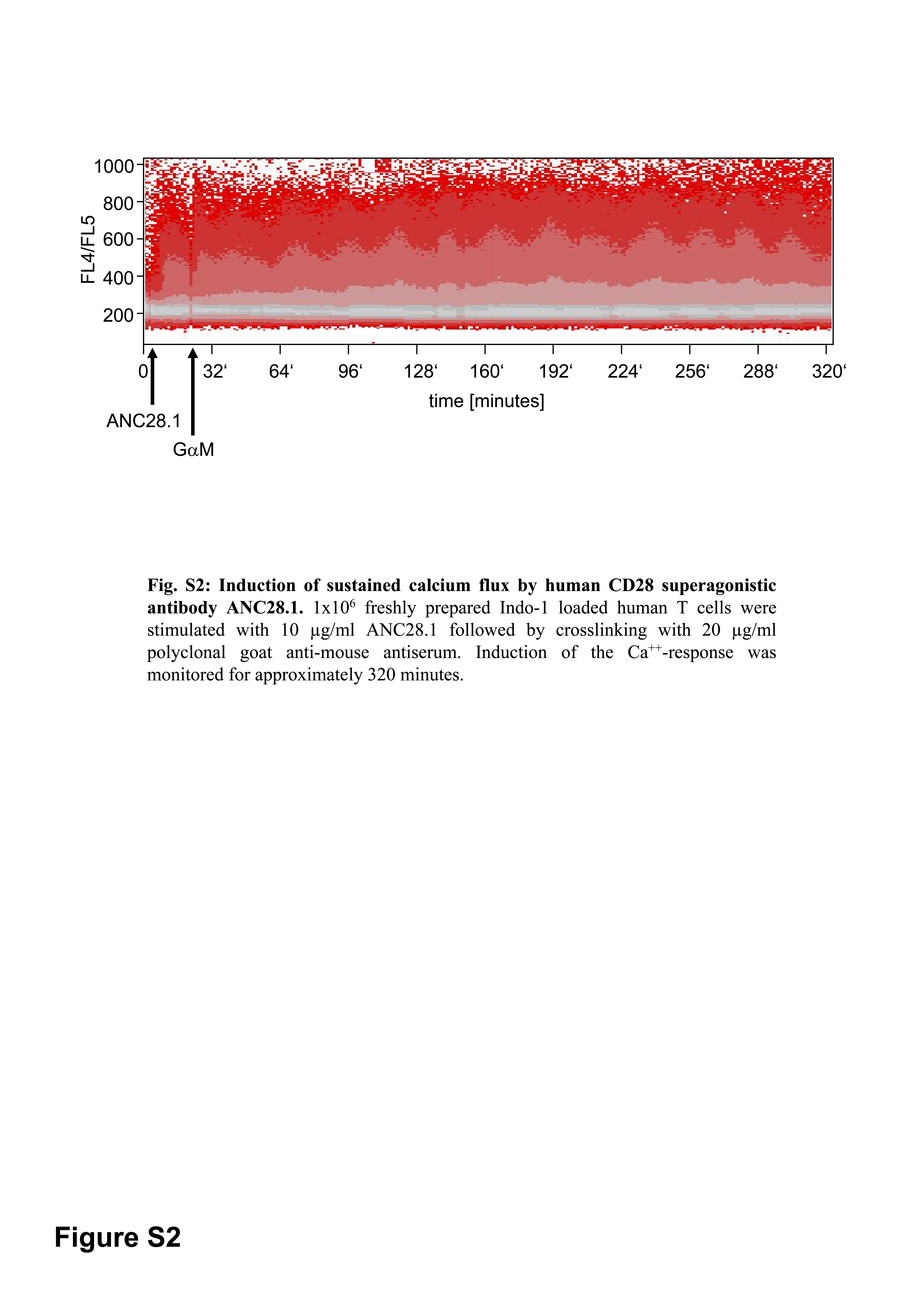

Supplement: Figure S2 — (3.22 MB TIF) [file pone.0001708.s002.tif]

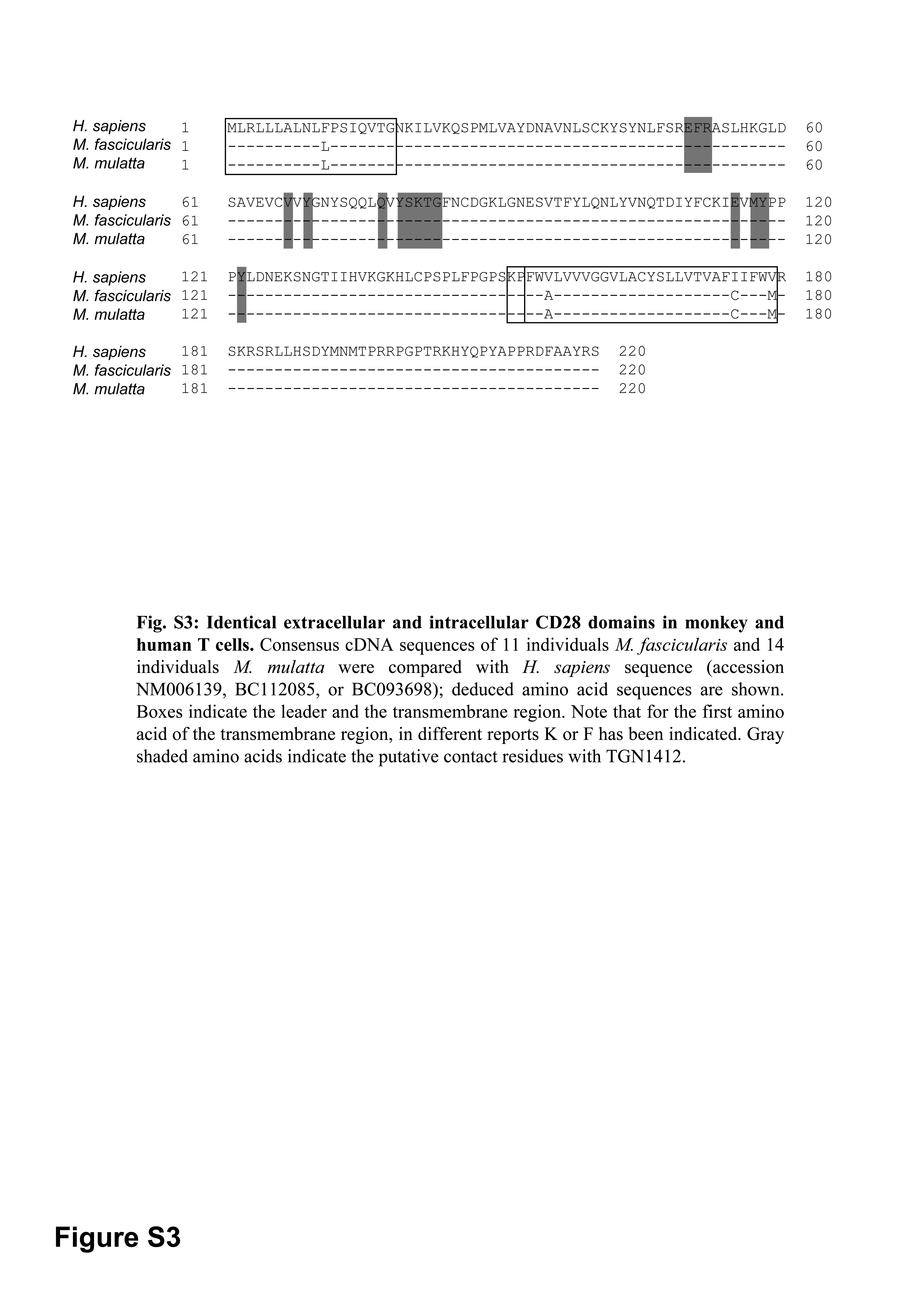

Supplement: Figure S3 — (0.51 MB TIF) [file pone.0001708.s003.tif]
